# Prevalence of graduate nurses with training in integrative and complementary health practices

**DOI:** 10.1590/0034-7167-2024-0318

**Published:** 2025-10-03

**Authors:** Daniela Dallegrave, Vitória Portarriaux Lopes Machado, Marluce Mechelli de Siqueira, Fabiana Gonring Xavier, Daiana Cristina Wickert, Calíope Pilger, Diéssica Roggia Piexak

**Affiliations:** I Universidade Federal do Rio Grande do Sul. Porto Alegre, Rio Grande do Sul, Brazil; II Universidade Federal do Rio Grande. Rio Grande, Rio Grande do Sul, Brazil; III Universidade Federal do Espírito Santo. Vitória, Espírito Santo, Brazil; IV Universidade Federal de Santa Maria. Santa Maria, Rio Grande do Sul, Brazil; V Universidade Federal de Catalão. Catalão, Goiás, Brazil

**Keywords:** Complementary Therapies, Nursing, Traditional Medicine Practitioners, Integrative Medicine, Public Health, Terapias Complementarias, Enfermería, Profesionales de Medicina Tradicional, Medicina Integradora, Salud Pública

## Abstract

**Objective:**

to demonstrate prevalence of graduate courses taken by nurses who have training in Integrative and Complementary Health Practices (ICHPs).

**Methods:**

a cross-sectional study, with data collection through a virtual questionnaire, with descriptive analysis.

**Results:**

a total of 1,154 nurses participated. It was found that the current scenario is predominantly composed of women (89.49%), with specialization or residency (57.45%), who dedicate 1 to 2 hours per week to the application of ICHPs, and 86.74% had a graduate degree in any area of knowledge. A significant association was identified between the possible presence of training in ICHPs, the presence of graduate degrees (p<0.001) and the level of graduate courses (p<0.001).

**Conclusions:**

a linear trend of increasing training in ICHPs was provided as the level of graduate degrees increased.

## RESUMEN

**Objetivo:**

demostrar la prevalencia de cursos de posgrado realizados por enfermeras con formación en Prácticas Integrativas y Complementarias de Salud (PICS).

**Métodos:**

estudio transversal, con recolección de datos a través de cuestionario virtual, con análisis descriptivo.

**Resultados:**

Participaron 1.154 enfermeras. Se encontró que el escenario actual está compuesto predominantemente por mujeres (89,49%), con especialización o residencia (57,45%), quienes dedican de 1 a 2 horas semanales a la aplicación del PICS, y el 86,74% tenía título de posgrado en alguna área del conocimiento. Se pudo identificar una asociación significativa entre la presencia de formación en PICS, la presencia de estudios de posgrado (p<0,001) y el nivel de cursos de posgrado (p<0,001).

**Conclusiones:**

se observó una tendencia lineal de aumento de la formación en PICS a medida que aumentaba el nivel de estudios de posgrado.

## INTRODUCTION

Integrative and Complementary Health Practices (ICHPs) are therapeutic approaches that aim to promote and restore health. These practices include evidence-based treatment techniques and methods as well as ancestral and cultural traditions. ICHPs emerge as a care focused on the complexity of the health-disease process, viewing users as a whole. Examples of ICHP include acupuncture, auriculotherapy, phytotherapy, homeopathy, among others^(1)^.

The Brazilian Health System (In Portuguese, *Sistema Único de Saúde* - SUS), through the Brazilian National Policy for Integrative and Complementary Practices (In Portuguese, *Política Nacional de Práticas Integrativas e Complementares* - PNPIC), recommends that the use of ICHPs be multidisciplinary, carried out mainly in the Primary Healthcare network^(2)^. Healthcare professions have regulated the use of ICHP in a corporate manner, i.e., through authorization acts that do not take into account multiprofessional integration^(3)^. Furthermore, it is observed that regulations that support professionals in the use of ICHPs are incipient, leading some authors to highlight that these professions seek legitimacy through state regulation^(3)^.

In the context of Brazilian nursing, ICHPs are regulated by the Federal Nursing Council (In Portuguese, *Conselho Federal de Enfermagem* - COFEN), which issued seven resolutions on the subject until 2023, four of which have been revoked. The remaining ones address professional practice regulation, especially in areas such as acupuncture and other individual care practices, requiring nurses to have specialized training^(4,5)^. In February 2024, COFEN Resolution 739 was published, presenting some definitions for training in the 29 practices contained in PNPIC, also supporting and directing the performance and training of nursing technicians and assistants, never mentioned in other resolutions^(6)^.

In order to understand some aspects related to the training profile of nurses seeking training in ICHP in Brazil, as well as, through the unprecedented analyses presented, contribute to the advancement of nursing in ICHP, the present research is justified.

## OBJECTIVE

To analyze prevalence of graduate courses taken by nurses who have training in ICHP.

## METHODS

### Ethical aspects

This research is a segment extracted from the macroproject ‘Inquérito Nacional sobre o Perfil Educacional e Profissional de Enfermeiros de Saúde Integrativa e Práticas Tradicionais - EnfPICS.’ The study was conducted in accordance with national ethical guidelines and received approval from the Universidade Federal do Rio Grande do Sul Research Ethics Committee. All participants provided informed consent online.

### Study Design, Location, and Period

This is a cross-sectional study, reported according to the Strengthening the Reporting of Observational studies in Epidemiology (STROBE) guidelines, and based on data from a national survey. The overall project employed a mixed-methods approach due to the complexity of the phenomenon; however, the present analysis focuses exclusively on quantitative data regarding the prevalence of graduate studies among nurses trained in ICHP.

However, it is worth highlighting that the analysis presented in this article focuses exclusively on quantitative data, aiming to highlight prevalence of graduate studies among nurses with training in ICHP.

Conducted throughout Brazil, the research aimed to outline the training and professional performance profile of nurses in ICHPs. Quantitative data collection was carried out through a virtual questionnaire from June 2021 to January 2022.

### Sample, inclusion and exclusion criteria

Based on a total population of 582,197 Brazilian nurses^(7)^, and using the sample calculation formula^(8)^, the minimum required sample size was 384 participants. The inclusion criterion was holding a nursing degree.

### Study protocol

The virtual questionnaire was administered via LimeSurvey at the Universidade Federal do Rio Grande do Sul and consisted of 52 questions. Seventeen questions were answered by all nurses (nine on sociodemographic profile and eight on professional profile). The remaining 34 questions were answered only by those who reported some training in ICHPs, covering topics such as general training, specific ICHP training, professional performance, and willingness to participate in the qualitative phase. The survey was disseminated via WhatsApp®, Facebook®, Instagram®, institutional pages, and email.

### Analysis of results, and statistics

The dependent variable of this study was training in ICHPs. Initially, the profile of participants was verified, observing: sex (female/male); age group quartiles; self-reported skin color (white, yellow, indigenous, brown, black); nationality (Brazilian, foreign); place of birth (by economic macroregions); marital status (married, divorced/separated, single, stable union/dating/living together, widowed); presence of children (no/yes); number of children (in numbers); time since graduation (in months); presence of a graduate course (no/yes) and its level; income (in minimum wages), etc. The influence of the presence of graduate education (no/yes) and its different levels (specialization/residency, academic master’s degree, professional master’s degree, academic/professional doctoral degree, post-doctoral degree) among study participants was investigated.

Stata IC version 16.0 was used for statistical analysis. First, descriptive analyses were performed, presenting the absolute and relative frequencies of each variable. The association between the presence of training in ICHPs and independent variables was analyzed using Pearson’s chi-square test. For the graduate level variable, p-value of linear trend was used to assess whether there was a hierarchy between the different categories. The effect measures assessed were Prevalence Ratio (PR), performed using Poisson Regression with robust adjustment for variance, with precision assessed by the 95% Confidence Interval (95%CI) and the significance of PR using the Wald test^(9)^. A significance level of less than 5% was adopted for all analyses in two-tailed tests. PRs were considered null when the unit value 1.0 was present in the 95%CI.

## RESULTS

### Participant characterization

A total of 1,154 nurses participated in the study. The mean age was 39.71 years (SD = 10.37 years). Of the participating nurses, 1,030 (89.49%) were female. Regarding nationality, 1,147 (99.65%) were Brazilian, of which 515 (45.06%) were born in the states of the southern macroregion (Paraná, Santa Catarina and Rio Grande do Sul) and almost half of participants (534; 46.36%) work in the states of the southern macroregion.

Among the nurses surveyed, 834 (72.65%) declared themselves white; 476 (41.28%) were married; and 657 (56.93%) had children, of whom 308 (47.24%) had only one child. A total of 1,004 (86.74%) participants declared having a religion. More than half of the participants (603; 52.44%) reported an income of up to four minimum wages. In relation to time since graduation, 336 (29.19%) professionals had completed their undergraduate degree more than 241 months ago (20 years) and 1.120 (97.31%) had an active registration with COFEN (Table 1).

**Table 1:** Factors associated with training in Integrative and Complementary Health Practices (N = 1,154)

	No training in ICHPs [n (%)]	With training in ICHPs [n (%)]	*p* value ^a^	*p* value Wald^c^	PR (95%CI)
**Graduate studies (n=1,154)**					
No (n=153)	113 (73.86%)	40 (26.14%)	P<0.001	P<0.001	1.0
Yes (n=1,001)	539 (53.85%)	462 (46.15%)			1.77 (1.34-2.32)
**Graduate level (n=1,154)**					
No graduate studies (n=154)	114 (74.03%)	40 (25.97%)	P<0.001^b^	P<0.001	1.0
Specialization/residency (n=663)	380 (57.32%)	283 (42.68%)			1.64 (1.24-2.18)
Academic master’s degree (n=144)	71 (49.31%)	73 (50.69%)			1.95 (1.43-2.67)
Professional master’s degree (n=60)	24 (40%)	36 (60%)			2.31 (1.65-3.24)
Academic/professional doctoral degree (n=107)	46 (42.99%)	61 (57.01%)			2.19 (1.64-3.24)
Post-doctoral degree (n=26)	17 (65.38%)	9 (34.62%)			1.33 (0.74-2.41)

Note:

a Pearson’s chi-square;

b linear trend p-value;

c Wald test; ICHPs - Integrative and Complementary Health Practices; PR - Prevalence Ratio; 95%CI - 95% Confidence Interval.

### Prevalence of training

Prevalence of training in ICHPs was 502/1,154 (43.50%). Approximately six out of seven participants had a graduate course in any area of knowledge, i.e., 1,001 (86.74%), with specialization/residency (663; 57.45%) being the most prevalent level. A significant association was observed between the presence of training in ICHPs, the presence of graduate studies (p<0.001) and the level of graduate courses (p<0.001).

Therefore, it is considered that having a graduate degree (regardless of the level) increases the occurrence of having a graduate degree increases the likelihood of receiving training in ICHPs by 77% (PR: 1.77; 95% CI: 1.34-2.32; p<0.001). With the exception of post-doctoral degree, a linear trend of increasing training in ICHPs was observed as the level of graduate degree increased, with participants with a professional master’s degree having a 131% (PR: 2.31; 95% CI: 1.65-3.24; p<0.001) greater probability of having training when compared to participants without graduate degree (Table 1).

Regarding the importance of training in ICHPs for professional life, 370 (73.85%) participants responded that they consider it very important. Concerning the performance and training in ICHP and the impact on the exercise of care activities, 489 (97.90%) responded that it had a positive impact. That said, 441 (88.20%) participants declared having greater autonomy in their profession.

As for the weekly hours dedicated to ICHP application, 119 (36.96%) participants responded that they dedicated 1 to 2 hours. Concerning the moment when participants became aware of ICHP as an area of nursing practice, 56.50% stated that they were not aware; 3.64% reported that they had knowledge before graduation; 10.22% reported that they had knowledge during graduation; 6.76% reported that they had knowledge at the beginning of their professional career; 17.85% reported that they had knowledge during their career; and 3.29% became aware of ICHP at the end of their professional career (Table 2).

**Table 2:** Knowledge about Integrative and Complementary Health Practices as a nursing area (N = 1,154)

	Graduate studies		Fisher’s exact test	Wald test	PR (95%CI)
	Não	Sim			
When did they learn about ICHP as a nursing area					
Was not aware	113 (17.33%)	539 (82.67%)	p<0.001	p<0.001	1.0
Before graduation	9 (21.43%)	33 (78.57%)			0.95 (0.81-1.17)
In graduation	20 (14.49%)	118 (85.51%)			1.03 (0.96-1.12)
At the beginning of their professional career	1 (1.28%)	77 (98.72%)			1.19 (1.14-1.25)
In the middle of their professional trajectory	6 (2.91%)	200 (97.09%)			1.17 (1.13-1.24)
At the end of their professional career	4 (10.53%)	34 (89.47%)			1.08 (0.97-1.21)

*Note: ICHP - Integrative and Complementary Health Practices; PR - Prevalence Ratio; 95%CI - 95% Confidence Interval.*

Regarding nurses who had knowledge about ICHPs during their undergraduate studies (N=138), 42.03% reported that they had taken a course on ICHPs; 36.96% reported that the subject was addressed within a course; 21.01% reported that they had listened to a lecture by a specialist; 15.22% reported having had knowledge through other means such as contact with people in the area and personal interest; 13.77% reported having participated in an extension project on ICHP; and 4.35% stated that they had participated as a scientific initiation scholarship holder in research on ICHPs.

In relation to the first contact with ICHP, this mostly happened as a user (15%); through the disclosure of a co-worker from another profession (11.79%); through the disclosure of people who are in the health area (9.36%); through other means such as family, personal search, movies, friends, etc. (6.67%); and through patient care (0.95%).

It was found that having a graduate course increases by 134% the possibility of a professional having knowledge about ICHP as a nursing area when compared to participants without a graduate degree (PR: 2.34; 95% CI: 1.70-3.22; p<0.001). However, in this last analysis, it is not possible to categorize according to the moment in which they became aware of ICHP as an area of training, as per the table above.

## DISCUSSION

Nursing has a significant workforce of 2,540,715 professionals, playing an important role in Brazilian health. This diverse community includes 438,886 nursing assistants, 1,476,584 nursing technicians and 624,910 nurses with academic training, taking over leadership, management and specialized care, influencing team coordination, clinical decisions and implementation of health policies^(10)^.

According to a previous study from 2022, there was a greater concentration of nurses in large urban centers and capitals; this pattern reflects a trend of professional agglomeration in metropolitan areas, characterized by population density and more developed health infrastructure. The preference for large urban centers can be attributed to several factors, such as better employment opportunities, access to advanced technological resources, greater diversity of specializations and opportunities to participate in research and development programs^(11)^.

A trend in the nursing scene in Brazil is the rejuvenation of the profession, characterized by the growing presence of younger professionals. This transformation appears to be driven by the substantial increase in the offer of courses, reflected in the increase in the number of graduates in the country in recent years. This situation points to a constant renewal in the demographic profile of nurses, with significant repercussions both on professional practice and on the composition of the workforce in the health area^(12)^.

Recent research linked to this macroproject, carried out specifically with nurses working in Santa Catarina, showed that the probability of an older nurse not seeking training in ICHPs was 17% higher than the probability of a younger nurse not seeking training, that is, an increase in the demand for training in ICHP can be expected^(13)^.

Thus, this change in the scenario of nursing professional training may be associated with a diversity in the Brazilian teaching model, in which of the 1,668 undergraduate nursing courses, 105 (6.29%) are offered in the Distance Learning (DL) modality, while 1,563 (93.70%) are in-person. The significant presence of in-person courses suggests an appreciation of in-person learning in nursing training, possibly related to the practical and interactive nature of the profession. However, the coexistence of DL courses, especially blended courses, points to a gradual adaptation of DL trends to meet students’ needs and educational market’s demands. This diversity of pedagogical approaches highlights the complexity of the educational scenario in the nursing field^(14)^.

In a study that aimed to analyze the general aspects of the nursing team’s work, it was found that approximately one third (31.4%) of nurses took a technical or assistant nursing course before completing their degree. Among the more than 130,000 nurses who underwent this additional training, it is notable that 86.1% of them claim to have actually practiced the activity. This finding reinforces the notion that these professionals have a solid professional experience even before completing their degree, corroborated by the fact that half of them (51%) were already in the job market before obtaining their degree^(12)^. This prior experience can have important implications for these nurses’ professional practice, offering a valuable and consolidated perspective in the field of nursing.

Regarding challenges related to training, 12.8% mention the lack of opportunities in their area of specialization, while 10.5% of respondents identify the lack of technical skills necessary for the field of work as a problem^(12)^.

Over the past 52 years, graduate nursing education in Brazil has seen an increase in the number of courses and programs available as well as an increase in the number of professionals trained. This growth reflects not only the maturation of the field of nursing as an area of study and practice, but also the growing appreciation of research and academic production^(15)^.

The graduate education system in Brazil comprises more than 4,500 programs in all areas of knowledge, of which about half grant the academic doctoral degree. This system is present in all regions of the country, with approximately 100,000 professors and annually graduating about 20,000 doctoral degree holders and 60,000 master’s degree holder^(16)^. According to a survey conducted by the Coordination for the Improvement of Higher Education Personnel in 2019, nursing is home to 54 academic graduate programs. Of these, 16 offer only master’s degrees; 36 offer both master’s and doctoral degrees; and two are dedicated exclusively to doctoral degrees, totaling 90 courses^(17)^.

In Brazil, the expansion of graduate programs in nursing was driven by the implementation of the Law of Guidelines and Bases of National Education, which establishes that undergraduate and graduate courses in professional education must be organized in accordance with national curricular guidelines defined by the Brazilian National Education Council in terms of objectives, characteristics and duration^(18)^. This measure made it possible to establish Higher Education Institutions designed to meet the needs of growth in the country’s technology sector. With the expansion of this process, there was a demand for qualified teachers to instruct new nursing professionals, leading to the creation of master’s and doctoral programs in several regions of Brazil^(19)^.

The increase in scientific production in high-impact journals suggests that nursing is gaining international recognition, consolidating itself as a robust and relevant field of study on the global stage. This can have positive effects not only for the profession itself, but also for the quality of healthcare offered to the population, based on increasingly solid and up-to-date evidence^(15)^. However, it is important to accompany this growth with guarantees of quality and excellence in professional training as well as with continuous investments in research and infrastructure to sustain this progress.

Concerning graduate education, this is an important tool capable of enhancing and improving technical-scientific knowledge, promoting the development of research skills, in addition to stimulating the deepening of knowledge about a specific field of study, exploring the chosen area in more detail. In this study, 86.74% of respondents reported having completed some type of graduate education, which supports another study carried out in 2022, with a total of 7,308 participants, which found that 80.1% had some graduate education^(20)^. In this comparison, it is observed that one in every five participants had graduate training^(20)^, while in the present study, this proportion was one in every seven participants.

As for the type of graduate studies undertaken, the findings of this study were greater in the residency and specialization types, in line with the COFEN study^(21)^, in which residency comprised a total of 7.5%, and specializations, 72.8%. A study^(20)^ that focused on nurses working in primary care found that 5.6% of nurses had completed graduate residency training and 73.3% had completed specialization training. It can be seen that there seems to be a greater demand for specializations, and this is also due to the greater supply of these.

Through the data from participants with graduate degrees presented in Table 1, it was possible to identify a directly proportional relationship in the increased chance of a professional having training in ICHPs when he or she already has some graduate degree.

When assessing the sociodemographic profile of nurses and analyzing which institutions were responsible for the academic training of these professionals, a study^(22)^ showed that 33.12% of individuals chose to develop their academic training in public higher education institutions, while 66.87% attended private institutions. This data is very similar to that found by authors^(23)^, in which it is possible to observe that there is a large concentration of nursing professionals (66.8%) who, during graduate studies, developed the specialization course in private institutions, while a third took it in public institutions.

Currently, there is a high percentage of professionals who have received training in ICHPs from short-term training courses, either in person or remotely, offered by the Ministry of Health, Municipal Health Departments and professional category councils. There are also those who graduated from private institutions, where the financial subsidy was paid for by the workers themselves, as they report not finding training courses for certain ICHPs in the public sector^(24)^.

In this context, it is shown that obtaining knowledge about integrative practices also occurs informally, in which professionals seek learning through social media, which reinforces the problem of the lack of educational actions for the qualification of professionals in the SUS. It is revealed that, among the obstacles reported by the participants, there are difficulties in the training offered by the Ministry of Health and/or State and Municipal Health Departments, with the reduced workload being among the main divergent points in training^(24)^.

This study showed that most professionals (17.85%) who have knowledge about ICHPs only learned about them as an area of professional activity during their career path, a difficulty also observed in a study^(25)^ in which nurses stated that they do not have such in-depth knowledge about ICHPs, since they did not obtain this knowledge during their undergraduate studies and do not have any updates on the subject. These professionals also report not having the physical structure and adequate resources to implement ICHPs in their workplaces.

This same difficulty was identified in another study involving 1,447 undergraduate students from six Brazilian universities, of whom only 300 (21.1%) were aware of PNPIC, while approximately 901 students (62.9%) admitted to not having knowledge about which integrative and complementary practices can be used in their professional area. Furthermore, 778 (56.1%) revealed that they did not have the necessary skills to guide, prescribe or refer people to use these practices. The study also revealed that 833 students (58.8%) were not aware of the interactions between these practices and conventional therapeutic treatments^(26)^.

Therefore, despite government efforts at all levels to promote health programs at national, state and municipal levels, ICHPs still face significant obstacles in their acceptance. These challenges may be linked to a number of factors, such as cultural resistance, lack of understanding about the benefits of ICHPs, regulatory issues, integration with conventional health practices, lack of mandatory courses on ICHPs in the academic curriculum of universities and the lack of opportunities for practical fields in institutions focused on offering ICHPs^(27)^. While ICHPs have the potential to offer a valuable complementary approach to healthcare, it is essential to address these barriers to ensuring their full acceptance and integration into health systems^(27)^. It is worth highlighting the importance of ICHPs in nurses’ perception of their professional autonomy when incorporating these practices into nursing consultations, especially with regard to problem-solving ability^(28)^.

However, the present study identified that, within the scope of nursing, professionals with knowledge of scientific knowledge and methods, which are conferred by graduate courses and diplomas, are those who migrate most to the area of ICHPs.

In this way, graduate training enriches the interest in the area of ICHPs, giving it a scientific character. This is because graduate studies contribute to the development of research in the health area, establishing a solid scientific basis and expanding knowledge.

### Study limitations

The survey was conducted virtually during the covid-19 pandemic, when nursing professionals were overwhelmed with work process demands. This situation may have influenced the number of respondents to the virtual survey and also the reach of participants from different regions of Brazil. Despite this, the sample was representative.

### Contributions to nursing and public policies

This research stands out for being a unique contribution to the field of ICHPs, by demonstrating how graduate studies strengthen engagement and provide an essential scientific basis for professionals working in ICHPs, using the example of nurses in this article. The research demonstrates, through its results, that graduate studies emerge as a fundamental catalyst for health research, consolidating scientific research in the work process of professionals who use ICHPs. Moreover, the research highlights the scarcity of literature, both national and international, on the training of nurses in these areas, which highlights a significant gap in scientific production to date.

## CONCLUSIONS

It was possible to analyze that completing a graduate course is associated with a greater probability of having training in ICHPs, and this result may be linked to professional interest, academic exposure and the search for constant updating that characterize this educational process.

However, it is important to strengthen training in ICHPs during the nursing undergraduate period, since most professionals only became aware of them during their professional careers. Therefore, this approach becomes essential to ensure that nursing professionals are adequately prepared to deal with the increasingly complex and diverse demands of the health system. This is essential to keep professionals current with best practices and to support a more integrative, evidence-based approach to healthcare

## Data Availability

Available research data is in the body of the article.
