# Supplementary material for: Prevalence of graduate nurses with training in integrative and complementary health practices
Source: Rev Bras Enferm. 2025 Oct 3;78(4):e20240318. doi: 10.1590/0034-7167-2024-0318 (PMC12494349; doi:10.1590/0034-7167-2024-0318)
Supplement: Translation

# Translation: Prevalência de pós-graduação em enfermeiros com formação em práticas integrativas e complementares em saúde

**DOI:** 10.1590/0034-7167-2024-0318pt

**Authors:** Daniela Dallegrave, Vitória Portarriaux Lopes Machado, Marluce Mechelli de Siqueira, Fabiana Gonring Xavier, Daiana Cristina Wickert, Calíope Pilger, Diéssica Roggia Piexak

**Affiliations:** I Universidade Federal do Rio Grande do Sul. Porto Alegre, Rio Grande do Sul, Brasil; II Universidade Federal do Rio Grande. Rio Grande, Rio Grande do Sul, Brasil; III Universidade Federal do Espírito Santo. Vitória, Espírito Santo, Brasil; IV Universidade Federal de Santa Maria. Santa Maria, Rio Grande do Sul, Brasil; V Universidade Federal de Catalão. Catalão, Goiás, Brasil

**Keywords:** Terapias Complementares, Enfermagem, Profissionais de Medicina Tradicional, Medicina Integrativa, Saúde Pública

## Abstract

**Objetivo:**

demonstrar a prevalência da realização de cursos de pós-graduação por enfermeiros que possuem formação em Práticas Integrativas e Complementares em Saúde (PICS).

**Métodos:**

estudo transversal, com coleta de dados por meio de questionário virtual, com análise descritiva.

**Resultados:**

participaram 1.154 enfermeiros. Constatou-se que o cenário atual é composto predominantemente por mulheres (89,49%), com especialização ou residência (57,45%), que dedicam de 1 a 2 horas semanais à aplicação das PICS, e 86,74% tinham um curso de pós-graduação em qualquer área do conhecimento. Foi possível identificar uma associação significativa entre a presença de formação em PICS, a presença de pós-graduação (p<0,001) e o nível dos cursos de pós-graduação (p<0,001).

**Conclusões:**

verificou-se uma tendência linear de aumento de formação em PICS conforme aumenta o nível de pós-graduação.

## INTRODUÇÃO

As Práticas Integrativas e Complementares em Saúde (PICS) são abordagens terapêuticas que visam à promoção e recuperação da saúde. Essas práticas incluem técnicas e métodos de tratamento baseados em evidências, bem como em tradições ancestrais e culturais. As PICS surgem como um cuidado voltado para a complexidade do processo saúde-doença, visualizando o usuário de modo integral. Exemplos de PICS incluem acupuntura, auriculoterapia, fitoterapia, homeopatia, entre outros^(1)^.

O Sistema Único de Saúde (SUS), por meio da Política Nacional de Práticas Integrativas e Complementares (PNPIC), preconiza que a utilização das PICS seja multiprofissional, realizada principalmente na rede de Atenção Primária à Saúde^(2)^. As profissões da saúde têm regulamentado o uso das PICS de maneira corporativa, isto é, através de atos autorizativos que não levam em consideração a integração multiprofissional^(3)^. Além disso, observa-se que as normativas que respaldam os profissionais para o uso das PICS são incipientes, levando alguns autores a destacar que essas profissões buscam legitimidade por meio da regulamentação estatal^(3)^.

No contexto da enfermagem brasileira, as PICS são regulamentadas pelo Conselho Federal de Enfermagem (COFEN), que emitiu, até 2023, sete resoluções sobre o tema, das quais quatro estão revogadas. As restantes abordam a regulamentação da atuação profissional, especialmente em áreas como acupuntura e outras práticas de cuidados individuais, exigindo dos enfermeiros formação em modalidade especializada^(4,5)^. Em fevereiro de 2024, foi publicada a Resolução COFEN nº 739, apresentando algumas definições para formação nas 29 práticas contidas na PNPIC, respaldando e direcionando também a atuação e formação do técnico e auxiliar de enfermagem, nunca mencionadas nas demais resoluções^(6)^.

No intuito de conhecer alguns aspectos relacionados ao perfil de formação dos enfermeiros que buscam a formação em PICS no Brasil, bem como, por meio das análises inéditas apresentadas, contribuir com o avanço da enfermagem nas PICS, justifica-se a presente pesquisa.

## OBJETIVO

Analisar a prevalência da realização de cursos de pós-graduação por enfermeiros que possuem formação em PICS.

## MÉTODOS

### Aspectos éticos

A pesquisa em questão é um segmento extraído do macroprojeto “Inquérito Nacional sobre o Perfil Educacional e Profissional de Enfermeiros de Saúde Integrativa e Práticas Tradicionais - EnfPICS”. Em termos éticos, o estudo foi conduzido de acordo com as diretrizes de ética nacionais, e recebeu aprovação do Comitê de Ética em Pesquisa da Universidade Federal do Rio Grande do Sul. Todas as pessoas que participaram do estudo registraram autorização no Termo de Consentimento Livre e Esclarecido por meio *online*.

### Desenho, local do estudo e período

Trata-se de estudo transversal, redigido com base no *STrengthening the Reporting of OBservational studies in Epidemiology* e realizado a partir de um inquérito nacional, o qual possui uma abordagem mista, tendo em vista a complexidade do fenômeno em análise e a importância de abordá-lo sob múltiplas perspectivas.

No entanto, vale ressaltar que a análise presente neste artigo se concentra exclusivamente em dados quantitativos, visando evidenciar a prevalência de pós-graduação em enfermeiros com formação em PICS.

Realizada em todo o território brasileiro, a pesquisa objetivou traçar o perfil de formação e atuação profissional de enfermeiros em PICS. A coleta de dados quantitativos foi realizada por meio de questionário virtual no período de junho de 2021 a janeiro de 2022.

### Amostra, critérios de inclusão e exclusão

Com base na população total, composta por 582.197 enfermeiros brasileiros^(7)^, e aplicando-se a fórmula do cálculo amostral^(8)^, chegou-se ao mínimo de 384 participantes. O critério de inclusão eram pessoas com diploma de graduação em enfermagem.

### Protocolo do estudo

O questionário virtual foi apresentado no *LimeSurvey* da Universidade Federal do Rio Grande do Sul, composto por 52 perguntas, sendo 17 para serem respondidas por todos os enfermeiros, divididas em nove questões relacionadas ao perfil sociodemográfico e, oito, ao perfil profissional. As demais, ou seja, 34 perguntas, foram respondidas especificamente por enfermeiros que afirmaram ter alguma formação em PICS, sendo duas sobre formação geral; 15 sobre formação em PICS (13 quantitativas e duas qualitativas); 17 sobre atuação profissional (12 quantitativas e cinco qualitativas); e a última questionava o participante sobre a disponibilidade de participar da etapa qualitativa do estudo. A divulgação da pesquisa se deu via *WhatsApp^®^, Facebook^®^, Instagram^®^*, páginas institucionais e e-mail.

### Análise dos resultados e estatística

A variável dependente deste estudo foi a formação em PICS. Inicialmente, foi verificado o perfil dos participantes, observando: o sexo (feminino/masculino); os quartis de faixa etária; a cor da pele autorreferida (branca, amarela, indígena, parda, preta); a nacionalidade (brasileira, estrangeira); a naturalidade (por macrorregiões econômicas); o estado civil (casado, divorciado/separado, solteiro, união estável/namorando/vivendo junto, viúvo); a presença de filhos (não/sim); o número de filhos (em números); o tempo de formado (em meses); a presença de curso de pós-graduação (não/sim) e o nível deste; a renda (em salários mínimos), *etc*. Foi investigada a influência da presença de pós-graduação (não/sim) e de seus diferentes níveis (especialização/residência, mestrado acadêmico, mestrado profissional, doutorado acadêmico/profissional, pós-doutorado) entre os participantes do estudo.

Para a análise estatística, utilizou-se o *software* Stata IC, versão 16.0. Em um primeiro momento, foram realizadas análises descritivas, apresentando as frequências absolutas e relativas de cada variável. A associação entre a presença de formação em PICS e as variáveis independentes foi analisada por meio do teste Qui-quadrado de Pearson. Para a variável nível de pós-graduação, foi utilizado o valor de *p* de tendência linear para avaliar se existia uma hierarquia entre as diferentes categorias. As medidas de efeito avaliadas foram a Razão de Prevalência (RP), executadas por meio de Regressão de Poisson com ajuste robusto para variância, com a precisão avaliada pelo Intervalo de Confiança de 95% (IC95%) e a significância da RP por meio do teste de Wald^(9)^. Foi adotado para todas as análises um nível de significância menor do que 5% em testes bicaudais. As RPs foram consideradas nulas quando o valor unitário 1,0 estava presente nos IC95%.

## RESULTADOS

### Caracterização dos participantes

Participaram do estudo 1.154 enfermeiros. A média de idade foi de 39,71 anos (DP=10,37 anos). Dos enfermeiros participantes, 1.030 (89,49%) eram do sexo feminino. A respeito da nacionalidade, 1.147 (99,65%) são brasileiros, sendo que 515 (45,06%) nasceram nos estados da macrorregião Sul (Paraná, Santa Catarina e Rio Grande do Sul) e quase a metade dos participantes (534; 46,36%) trabalha nos estados da macrorregião Sul.

Entre os enfermeiros pesquisados, 834 (72,65%) se autodeclararam brancos; 476 (41,28%) eram casados; e 657 (56,93%) tinham filhos, sendo que, entre estes, 308 (47,24%) tinham apenas um filho. Declararam ter uma religião 1.004 (86,74%) participantes. Mais da metade dos participantes (603; 52,44%) afirmou ter como renda até quatro salários mínimos. Quanto ao tempo de formação, 336 (29,19%) profissionais tinham concluído a graduação há mais de 241 meses (20 anos) e 1.120 (97,31%) tinham registro ativo no COFEN (Tabela 1).

**Tabela 1:** Fatores associados à formação em Práticas Integrativas e Complementares em Saúde (N = 1.154)

	Sem formação em PICS [n (%)]	Com formação em PICS [n (%)]	Valor de *p* ^a^	Valor de *p* Wald^c^	RP (IC95%)
**Pós-graduação (n=1.154)**					
Não (n=153)	113 (73,86%)	40 (26,14%)	P<0,001	P<0,001	1,0
Sim (n=1.001)	539 (53,85%)	462 (46,15%)			1,77 (1,34-2,32)
**Nível de pós-graduação (n=1.154)**					
Sem pós-graduação (n=154)	114 (74,03%)	40 (25,97%)	P<0,001^b^	P<0,001	1,0
Especialização/residência (n=663)	380 (57,32%)	283 (42,68%)			1,64 (1,24-2,18)
Mestrado acadêmico (n=144)	71 (49,31%)	73 (50,69%)			1,95 (1,43-2,67)
Mestrado profissional (n=60)	24 (40%)	36 (60%)			2,31 (1,65-3,24)
Doutorado acadêmico/profissional (n=107)	46 (42,99%)	61 (57,01%)			2,19 (1,64-3,24)
Pós-doutorado (n=26)	17 (65,38%)	9 (34,62%)			1,33 (0,74-2,41)

Nota:

a qui-quadrado de Pearson;

b valor de p de tendência linear;

c teste de Wald; PICS - Práticas Integrativas e Complementares em Saúde; RP - Razão de Prevalência; IC95% - Intervalo de Confiança de 95%.

### Prevalência de formação

A prevalência de formação em PICS foi de 502/1.154 (43,50%). Aproximadamente seis a cada sete participantes tinham um curso de pós-graduação em qualquer área do conhecimento, isto é, 1.001 (86,74%), sendo a especialização/residência (663; 57,45%) o nível mais prevalente. Observou-se uma associação significativa entre a presença de formação em PICS, a presença de pós-graduação (p<0,001) e o nível dos cursos de pós-graduação (p<0,001).

Considera-se, portanto, que ter uma pós-graduação (independentemente do nível) aumenta a ocorrência de ter formação em PICS em 77% (RP: 1,77; IC95%:1,34-2,32; p<0,001). Com exceção do pós-doutorado, foi constatada uma tendência linear de aumento de formação em PICS conforme aumentava o nível de pós-graduação, sendo que os participantes com mestrado profissional apresentaram probabilidade de 131% (RP: 2,31; IC95%:1,65-3,24; p<0,001) a mais de terem formação, quando comparado com participantes sem pós-graduação (Tabela 1).

Quanto à importância da formação em PICS para a vida profissional, 370 (73,85%) participantes responderam que acham muito importante. No que concerne à atuação e formação em PICS e à repercussão no exercício da atividade assistencial, 489 (97,90%) responderam que tiveram uma repercussão positiva. Posto isto, 441 (88,20%) participantes declararam ter maior autonomia na sua profissão.

Acerca das horas semanais dedicadas à aplicação de PICS, 119 (36,96%) participantes responderam dedicar-se de 1 a 2 horas. Quanto ao momento em que os participantes tiveram conhecimento sobre as PICS como área de atuação do enfermeiro, 56,50% declararam que não tomaram conhecimento; 3,64% informaram que tiveram conhecimento antes da graduação; 10,22% informaram que tiveram conhecimento na graduação; 6,76% informaram que tiveram conhecimento no início da trajetória profissional; 17,85% informaram que tiveram conhecimento no decurso da trajetória; e 3,29% tiveram conhecimento das PICS no final da trajetória profissional (Tabela 2).

**Tabela 2:** Conhecimento sobre as Práticas Integrativas e Complementares em Saúde como área do enfermeiro (N = 1.154)

	Pós-graduação		Teste exato de Fisher	Teste de Wald	RP (IC95%)
	Não	Sim			
Quando tomou conhecimento sobre as PICS como área do enfermeiro					
Não tomou conhecimento	113 (17,33%)	539 (82,67%)	p<0,001	p<0,001	1,0
Antes da graduação	9 (21,43%)	33 (78,57%)			0,95 (0,81-1,17)
Na graduação	20 (14,49%)	118 (85,51%)			1,03 (0,96-1,12)
No início da trajetória profissional	1 (1,28%)	77 (98,72%)			1,19 (1,14-1,25)
No meio da trajetória profissional	6 (2,91%)	200 (97,09%)			1,17 (1,13-1,24)
No final da trajetória profissional	4 (10,53%)	34 (89,47%)			1,08 (0,97-1,21)

*Nota: PICS - Práticas Integrativas e Complementares em Saúde; RP - Razão de Prevalência; IC95% - Intervalo de Confiança de 95%.*

Quanto aos enfermeiros que tiveram conhecimento sobre as PICS durante a graduação (N=138), 42,03% informaram que cursaram uma disciplina sobre PICS; 36,96% relataram que o assunto foi abordado dentro de uma disciplina qualquer; 21,01% informaram que ouviram uma palestra com profissional especialista; 15,22% relataram ter tido conhecimento através de outros meios como contato com pessoas da área e interesse pessoal; 13,77% informaram ter participado de projeto de extensão sobre as PICS; e 4,35% declararam que participaram como bolsista de iniciação científica em pesquisa sobre PICS.

Quanto ao primeiro contato com as PICS, majoritariamente isso aconteceu como usuário (15%); por meio da divulgação de algum colega de outra profissão (11,79%); por meio da divulgação de pessoas que são da área da saúde (9,36%); por outros meios como a família, a busca pessoal, filmes, amigos, *etc*. (6,67%); e por meio do atendimento de um paciente (0,95%).

Constata-se que ter um curso de pós-graduação aumenta em 134% a possibilidade de o profissional ter conhecimento sobre as PICS como área do enfermeiro, quando comparado aos participantes sem pós-graduação (RP:2,34; IC95%:1,70-3,22; p<0,001). No entanto, nesta última análise, não é possível categorizar de acordo com o momento em que tomou o conhecimento das PICS como área de formação, conforme tabela acima.

## DISCUSSÃO

A enfermagem conta com força de trabalho expressiva de 2.540.715 profissionais, desempenhando importante papel na saúde brasileira. Essa comunidade diversificada inclui 438.886 auxiliares de enfermagem, 1.476.584 técnicos de enfermagem e 624.910 enfermeiros com formação acadêmica, assumindo liderança, gestão e cuidados especializados, influenciando a coordenação de equipes, decisões clínicas e implementação de políticas de saúde^(10)^.

De acordo com estudo anterior, de 2022, houve uma maior concentração de enfermeiros nos grandes centros urbanos e capitais; este padrão reflete uma tendência de aglomeração profissional em áreas metropolitanas, caracterizadas pela densidade populacional e infraestrutura de saúde mais desenvolvida. A preferência por grandes centros urbanos pode ser atribuída a diversos fatores, como melhores oportunidades de emprego, acesso a recursos tecnológicos avançados, maior diversidade de especializações e oportunidades de participação em programas de pesquisa e desenvolvimento^(11)^.

Uma tendência no cenário da enfermagem no Brasil é o rejuvenescimento na profissão, caracterizado pela crescente presença de profissionais mais jovens. Essa transformação parece ser impulsionada pelo substancial aumento na oferta de cursos, refletido no aumento do número de graduados no país nos últimos anos. Essa conjuntura aponta para uma constante renovação no perfil demográfico dos enfermeiros, com repercussões significativas tanto na prática profissional quanto na composição da força de trabalho na área da saúde^(12)^.

Recente pesquisa vinculada ao presente macroprojeto, realizada especificamente com enfermeiros atuantes em Santa Catarina, evidenciou que a probabilidade de um enfermeiro com idade elevada não procurar a formação em PICS foi 17% maior do que a probabilidade de um enfermeiro mais jovem não buscar a formação, ou seja, pode ser esperado um aumento da procura pela formação em PICS^(13)^.

Desse modo, essa mudança no cenário da formação de profissionais enfermeiros pode estar associada a uma diversidade no modelo de ensino brasileiro, no qual dos 1.668 cursos de graduação em enfermagem, 105 (6,29%) são oferecidos na modalidade de Ensino a Distância (EaD), enquanto 1.563 (93,70%) são presenciais. A presença significativa de cursos presenciais sugere uma valorização do aprendizado presencial na formação em enfermagem, possivelmente relacionado à natureza prática e interativa da profissão. No entanto, a coexistência de cursos EaD, especialmente semipresenciais, aponta para uma adaptação gradual das tendências de EaD para atender às necessidades dos estudantes e às demandas do mercado educacional. Essa diversidade de abordagens pedagógicas destaca a complexidade do cenário educacional na área da enfermagem^(14)^.

Em estudo que objetivou analisar os aspectos gerais do trabalho da equipe de enfermagem, foi verificado que aproximadamente um terço (31,4%) dos enfermeiros realizou curso técnico ou auxiliar de enfermagem antes de concluir sua graduação. Entre os mais de 130 mil enfermeiros que passaram por essa formação adicional, notável é o fato de que 86,1% deles afirmam ter exercido efetivamente a atividade. Essa constatação reforça a noção de que esses profissionais possuem uma sólida vivência profissional antes mesmo de concluir sua graduação, corroborada pelo fato de que metade deles (51%) já estava inserida no mercado de trabalho antes de obter o diploma^(12)^. Essa experiência prévia pode ter implicações importantes para a prática profissional desses enfermeiros, oferecendo uma perspectiva valiosa e consolidada no campo da enfermagem.

No que diz respeito aos desafios relacionados à formação, 12,8% mencionam a carência de oportunidades na sua área de especialização, enquanto que 10,5% dos entrevistados identificam como problema a falta de habilidades técnicas necessárias para o campo de trabalho^(12)^.

A pós-graduação em enfermagem no Brasil, ao longo de 52 anos, tem evidenciado um aumento no número de cursos e programas disponíveis, bem como um aumento na quantidade de profissionais formados. Esse crescimento reflete não apenas o amadurecimento do campo da enfermagem como uma área de estudo e prática, mas também a crescente valorização da pesquisa e produção acadêmica^(15)^.

O sistema educacional de pós-graduação no Brasil compreende mais de 4.500 programas em todas as áreas do conhecimento, dos quais cerca de metade concede o título de doutorado acadêmico. Este sistema está presente em todas as regiões do país, contando com aproximadamente 100.000 professores e formando anualmente cerca de 20.000 doutores e 60.000 mestres^(16)^. Segundo um levantamento realizado pela Coordenação de Aperfeiçoamento de Pessoal de Nível Superior em 2019, o campo da enfermagem abriga 54 programas de pós-graduação acadêmica. Dentro desses, 16 oferecem somente cursos de mestrado; 36 proporcionam tanto cursos de mestrado quanto de doutorado; e dois se dedicam exclusivamente ao doutorado, totalizando 90 cursos^(17)^.

No Brasil, a ampliação dos programas de pós-graduação em enfermagem foi impulsionada pela implementação da Lei de Diretrizes e Bases da Educação Nacional, que estabelece que os cursos de graduação e pós-graduação em educação profissional devem ser organizados de acordo com as diretrizes curriculares nacionais definidas pelo Conselho Nacional de Educação em termos de objetivos, características e duração^(18)^. Essa medida possibilitou o estabelecimento das Instituições de Ensino Superior destinadas a suprir as necessidades de crescimento no setor tecnológico do país. Com a expansão desse processo, houve uma demanda de professores qualificados para instruir os novos profissionais de enfermagem, levando à criação de programas de mestrado e doutorado em várias regiões do Brasil^(19)^.

O aumento na produção científica em periódicos de alto impacto sugere que a enfermagem está ganhando reconhecimento internacional, consolidando-se como um campo de estudo robusto e relevante no cenário global. Isso pode ter efeitos positivos não apenas para a profissão em si, mas também para a qualidade dos cuidados de saúde oferecidos à população, com base em evidências cada vez mais sólidas e atualizadas^(15)^. No entanto, é importante acompanhar esse crescimento com garantias de qualidade e excelência na formação dos profissionais, bem como com investimentos contínuos em pesquisa e infraestrutura para sustentar esse avanço.

Em relação à formação pós-graduada, esta é uma importante ferramenta capaz de potencializar e aprimorar os conhecimentos técnico-científicos, promovendo o desenvolvimento de habilidades de pesquisa, além de estimular o aprofundamento do conhecimento acerca de um campo de estudo específico, explorando de forma mais detalhada a área escolhida. Neste estudo, 86,74% dos respondentes informaram ter realizado algum tipo de pós-graduação, o que corrobora outro estudo realizado em 2022, com um total de 7.308 participantes, que verificaram que 80,1% possuíam alguma pós-graduação^(20)^. Nessa comparação, observa-se que um a cada cinco participantes possuíam formação na modalidade pós-graduação^(20)^, enquanto que, no presente estudo, essa proporção foi de um a cada sete participantes.

No que se refere ao tipo de pós-graduação realizada, os achados deste estudo foram maiores nos tipos residência e especialização, indo ao encontro do estudo do COFEN^(21)^, no qual a residência compôs um total de 7,5%, e as especializações, 72,8%. Estudo^(20)^ que teve como foco os enfermeiros atuantes na atenção básica constatou que 5,6% dos enfermeiros haviam realizado pós-graduação na modalidade residência e, 73,3%, na modalidade especialização. Pode-se observar que parece haver uma procura maior pelas especializações, e isso se deve também pela maior oferta das mesmas.

Por meio dos dados dos participantes com pós-graduação apresentados na Tabela 1, foi possível identificar uma relação diretamente proporcional no aumento da chance de o profissional ter formação em PICS quando este já possui alguma pós-graduação.

Avaliando o perfil sociodemográfico de enfermeiros e analisando quais instituições foram responsáveis pela formação acadêmica desses profissionais, estudo^(22)^ apontou que 33,12% dos indivíduos optaram por desenvolver sua formação acadêmica em instituições públicas de ensino superior, enquanto que 66,87% cursaram em instituições privadas. Esse dado é muito semelhante ao encontrado por autores^(23)^, em que é possível observar que há uma grande concentração de profissionais de enfermagem (66,8%) que, durante a pós-graduação, desenvolveram o curso de especialização em instituições privadas, enquanto que um terço realizou em instituições públicas.

Atualmente, existe um percentual elevado de profissionais que possuem formação em PICS a partir de cursos de capacitação de curta duração, de modo presencial ou a distância, oferecidos pelo Ministério da Saúde, Secretarias Municipais de Saúde e conselhos de categorias profissionais. Há, também, aqueles que se formaram em instituições privadas, em que o subsídio financeiro foi custeado pelo próprio trabalhador, pois relatam não encontrarem cursos de capacitação de determinadas PICS no setor público^(24)^.

Nesta conjuntura, retrata-se que a obtenção de conhecimento em torno das práticas integrativas se dá, também, de modo informal, em que os profissionais buscam aprendizagem através das mídias sociais, o que reforça a problemática da carência de ações educativas para a qualificação dos profissionais no SUS. Revela-se que, entre os obstáculos relatados pelos participantes, encontram-se dificuldades nas capacitações oferecidas pelo Ministério da Saúde e/ou Secretarias Estaduais e Municipais de Saúde, sendo que a carga horária reduzida está entre os principais pontos divergentes na formação^(24)^.

Evidenciou-se, neste estudo, que a maioria dos profissionais (17,85%) que tem conhecimento acerca das PICS só tomou conhecimento sobre elas como área de atuação profissional durante seu itinerário no mundo do trabalho, dificuldade também observada em pesquisa^(25)^ na qual enfermeiras afirmaram que não possuem um conhecimento tão aprofundado acerca das PICS, visto que não obtiveram este conhecimento na sua graduação e nem possuem atualizações sobre o tema. Essas profissionais, também, relatam não dispor de estrutura física e recursos adequados para implementação das PICS em seus locais de trabalho.

Essa mesma dificuldade é identificada em outra pesquisa que contou com a participação de 1.447 universitários graduandos de seis universidades brasileiras, dos quais apenas 300 (21,1%) tinham conhecimento sobre a PNPIC, sendo que cerca de 901 alunos (62,9%) admitiram não ter conhecimento sobre quais práticas integrativas e complementares podem ser utilizadas em sua área profissional. Além disso, 778 (56,1%) revelaram não possuir habilidades necessárias para orientar, prescrever ou encaminhar pessoas para o uso dessas práticas. A pesquisa também revelou que 833 alunos (58,8%) não tinham conhecimento sobre as interações entre essas práticas e os tratamentos terapêuticos convencionais^(26)^.

Portanto, apesar dos esforços governamentais em todos os níveis para promover programas de saúde em âmbito nacional, estadual e municipal, as PICS ainda enfrentam obstáculos significativos em sua aceitação. Esses desafios podem estar ligados a uma série de fatores, como resistência cultural, falta de compreensão sobre os benefícios das PICS, questões de regulamentação, integração com práticas convencionais de saúde, carência de disciplinas obrigatórias sobre as PICS no currículo acadêmico das universidades e a falta de oportunidade de campos práticos em instituições voltadas para a oferta das PICS^(27)^. Embora as PICS tenham o potencial de oferecer uma abordagem complementar valiosa para o cuidado da saúde, é essencial enfrentar esses obstáculos para garantir sua plena aceitação e integração nos sistemas de saúde^(27)^. Vale destacar a importância das PICS na percepção dos enfermeiros sobre a sua autonomia profissional ao incorporar essas práticas na consulta de enfermagem, especialmente no que se refere à resolutividade^(28)^.

Contudo, o presente estudo identificou que, no âmbito da enfermagem, os profissionais com conhecimento dos saberes e métodos de cunho científico, os quais são conferidos pelos cursos e diplomas de pós-graduação, são os que mais migram para a área das PICS.

Dessa forma, a formação em nível de pós-graduação enriquece o interesse na área das PICS, conferindo um caráter científico. Isso ocorre porque a pós-graduação contribui para o desenvolvimento de pesquisas na área da saúde, estabelecendo uma base científica sólida e ampliando o conhecimento.

### Limitações do estudo

A pesquisa foi realizada de modo virtual durante o período da pandemia de covid-19, no qual os profissionais de enfermagem estavam sobrecarregados com demandas do processo de trabalho. Essa situação pode ter influenciado o número de respondentes do questionário virtual e também o alcance de participantes oriundos das diversas regiões brasileiras. Apesar disso, a amostra foi representativa.

### Contribuições para as áreas da enfermagem e políticas públicas

A presente pesquisa destaca-se por tratar-se de uma contribuição singular para o campo das PICS, ao demonstrar como a pós-graduação fortalece o engajamento e confere um embasamento científico essencial aos profissionais que atuam nas PICS, usando o exemplo dos enfermeiros neste artigo. A pesquisa demonstra, por meio de seus resultados, que a pós-graduação emerge como um catalisador fundamental para a pesquisa em saúde, consolidando a busca científica no processo de trabalho de profissionais que usam as PICS. Além disso, a pesquisa evidencia a escassez de literatura, tanto nacional quanto internacional, sobre a formação de enfermeiros nessas áreas, o que ressalta uma lacuna marcante na produção científica até o presente momento.

## CONCLUSÕES

Foi possível analisar que a realização de uma pós-graduação está associada a uma maior probabilidade de ter formação em PICS, podendo esse resultado estar vinculado ao interesse profissional, exposição acadêmica e busca por atualização constante que caracterizam esse processo educacional.

Contudo, evidencia-se a importância em fortalecer a formação em PICS durante o período de graduação em enfermagem, visto que a maioria dos profissionais só teve conhecimento das mesmas no decurso da trajetória profissional. Sendo assim, esta abordagem se torna essencial para garantir que os profissionais de enfermagem estejam devidamente preparados para lidar com as demandas cada vez mais complexas e diversificadas do sistema de saúde. Isso é crucial para manter os profissionais atualizados com as práticas mais recentes e promover uma abordagem mais integrativa e cientificamente embasada no cuidado à saúde.

## Data Availability

Os dados de pesquisa disponíveis estão no corpo do artigo.
